# Reconsidering the meaning of ‘stiff’ in clinical assessment of foot joint mobility

**DOI:** 10.1186/1757-1146-8-S2-O17

**Published:** 2015-09-22

**Authors:** Marabelle L Heng, Pui W Kong

**Affiliations:** 1 Podiatry Department, Singapore General Hospital, Singapore 169608; 2 Physical Education & Sports Science Academic Group, National Institute of Education, Nanyang Technological University, Singapore 637616

**Keywords:** Force, displacement, stiffness, hypermobility

## Background

Hypermobile joints in the medial column of the foot are associated with conditions such as hallux valgus and metatarsalgia. While hypermobility is often regarded as “an excessive dorsal excursion with a soft end-point of motion”, clinicians and scientists have not come to a consensus on how foot joint mobility should be assessed.

## Process

A literature search was done to review how joint mobility in the medial column of the foot was assessed. Keywords used included ‘foot’, ‘medial column’, ‘first ray’, ‘first metatarsophalangeal joint’, ‘hypermobility’, ‘stiffness’ and ‘assessment’.

## Findings

There are several ways to assess foot joint mobility:

(i) Subjective rating - a joint is moved through its range of motion by a tester who then grades the joint as ‘stiff’, ‘normal’ or ‘hypermobile’.

(ii) Maximum range of motion (ROM) - measured using goniometry and ruler scales.

(iii) Joint stiffness – joint displacement and force applied are taken into account to calculate joint stiffness. In the foot, only the stiffness for the first ray has been reported in literature.

Subjective rating is dependent on the tester's experience and not reliable. The subjective rating of a ‘stiff’ joint is often confused with the mechanical definition of ‘stiffness’ which is calculated as “force divided by displacement”. While maximum ROM is commonly used as a proxy of joint mobility, this method does not accurately reflect joint stiffness since the force applied is not considered. For instance, two subjects may have the same maximum ROM in their first metatarsophalangeal joint but the force required to displace the joint can be different (i.e. the joint stiffness is different). Unfortunately, there is no commercially available equipment to capture foot joint displacement and the associated force. Hence, joint stiffness is rarely measured in clinical settings.

## Conclusions

When assessing joint mobility in the foot, one should consider both joint displacement and the force applied to displace a joint. There is a need to develop clinically friendly protocols to assess foot joint stiffness objectively.

